# The impact of sexual violence in mid-adolescence on mental health: a UK population-based longitudinal study

**DOI:** 10.1016/S2215-0366(22)00271-1

**Published:** 2022-11

**Authors:** Francesca Bentivegna, Praveetha Patalay

**Affiliations:** a UCL Institute of Education, Department of Psychology and Human Development; University College London, London, UK; b Centre for Longitudinal Studies, Social Research Institute; University College London, London, UK; c MRC Unit for Lifelong Health and Ageing; University College London, London, UK

## Abstract

**Background:**

A large gender gap appears in internalising mental health conditions during adolescence, with higher rates in girls than boys. There is little high-quality longitudinal population-based research investigating the role of sexual violence experiences, which are disproportionately experienced by girls. We aimed to estimate the effects of sexual violence experienced in mid-adolescence on mental health outcomes.

**Methods:**

In this study, we used data from the longitudinal UK Millennium Cohort Study, a large nationally representative cohort of children born in the UK in 2000–02, for participants with information available at age 17 years on sexual violence in the past year (eg, sexual assault or unwelcome sexual approach), mental health outcomes (eg, completion of the Kessler Psychological Distress K6 scale in the past 30 days, self-harm in the past year, and lifetime attempted suicide). Multivariable confounder adjusted regressions and propensity matching approaches were used, and population attributable fractions (PAFs) were calculated.

**Findings:**

We included 5119 girls and 4852 boys (8063 [80·8%] of whom were White) in the full analysis sample. In the fully adjusted model, compared with no sexual violence, sexual violence was associated with greater mean psychological distress in girls (mean difference 2·09 [95% CI 1·51–2·68]) and boys (2·56 [1·59–3·53]), higher risk of high psychological distress in girls (risk ratio [RR] 1·65 [95% CI 1·37–2·00]) and boys (1·55 [1·00–2·40]), higher risk of self-harm in girls (RR 1·79 [1·52–2·10]) and boys (RR 2·16 [1·63–2·84]), and higher risk of attempted suicide in girls (RR 1·75 [1·26–2·41]) and boys (RR 2·73 [1·59–4·67]). PAF estimates suggest that, in a hypothetical scenario with no sexual violence, the prevalence of adverse mental health outcomes at age 17 years would be 3·7–10·5% lower in boys and 14·0–18·7% lower in girls than the prevalence in this cohort.

**Interpretation:**

Reductions in sexual violence via policy and societal changes would benefit the mental health of adolescents and might contribute to narrowing the gender gap in internalising mental ill health. Clinicians and others working to support adolescents should be aware that sexual violence has a widespread, gendered nature and an impact on mental health.

**Funding:**

UK Medical Research Council.

## Introduction

There is a well established and substantial gender gap in the prevalence of common mental illnesses. Women and girls have higher rates of depression, anxiety, and self-harm than men and boys from adolescence onwards.^1^, ^2^ There are many possible explanations for this gender gap, including different social risks, biological development, and specific vulnerability factors (eg, cognitive style, emotional reactivity, and genetic vulnerability) in interaction with stressors that are particularly salient in adolescence.^3^, ^4^ It is probable that several factors contribute to the larger burden of these mental health problems faced by women and girls than by men and boys. One potential risk factor that is disproportionately experienced by females from early adolescence is sexual violence, including sexual abuse, assault, and harassment.^5^ Teenage girls are more than five times more likely to experience sexual assault than their male peers.^6^ Estimating the effect of sexual violence in mid-adolescence (ie, between 14 and 18 years of age) on subsequent mental health is, therefore, crucial.^7^

The available evidence on the effect of sexual violence in mid-adolescence on mental health is scarce, because many studies evaluating the effects of sexual violence before adulthood have focused on adverse childhood experiences (including sexual abuse earlier in childhood^8^, ^9^), experiences of sexual violence in the university and college environment,^10^, ^11^ and intimate partner violence in older adolescent age groups.^12^ Several studies we identified that focused on mid-adolescence—during which many individuals begin to experience increasing rates of sexual harassment and assault after puberty—cannot make assumptions on the population-level effects of sexual violence due to the use of selective samples (eg, samples recruited from sexual-assault referral centres^13^), the examination of cross-sectional associations,^14^, ^15^ or the use of retrospective recollections of both the violence experienced and its perceived subsequent effect on mental health.^7^, ^16^ Previous studies with longitudinal examination have been limited by the use of convenience samples in some North American school settings.^17^, ^18^ Furthermore, these studies did not include factors (eg, socioeconomic circumstances, prior mental health, family structure, health, and sexuality) that might be confounders of the association between sexual violence and mental health outcomes.^19^, ^20^

**Research in context**

**Evidence before this study**

PubMed was searched using relevant search terms related to sexual violence, mental health, and adolescence in the English language (appendix p 23) from database inception to Dec 1, 2021, alongside searches in Google Scholar and via reference pearling (ie, searching through references of papers to find more relevant references). We found cross-sectional studies and studies in clinical samples, which are not suitable for understanding the population-level effects of sexual violence on the mental health of adolescents. A few longitudinal studies in convenience school-based samples were also identified, the generalisability of which is limited to the populations in these contexts, and they did not include relevant wider and earlier life factors that might be important confounders of the relationship between sexual violence and mental health. Previous evidence suggests that sexual violence in adolescence is associated with greater mental health difficulties; however, the design of these studies precludes estimates that can be translated to population-level effects.

**Added value of this study**

Using a rich, longitudinal, population-based cohort sample drawn to be representative of individuals born in the UK at the start of the 2000s, we estimated the effect of sexual violence experiences in mid-adolescence on mental ill health. We found that both girls and boys who experienced sexual violence in the past 12 months reported greater rates of psychological distress, self-harm, and attempted suicide at age 17 years compared with those without these experiences. These effects persisted after accounting for previous psychological distress and self-harm and a wide range of relevant confounders. Population-attributable fractions indicate that 14·0–18·7% of mental health difficulties in girls and 3·7–10·5% of mental health difficulties in boys at age 17 years were attributable to sexual violence experiences.

**Implications of all the available evidence**

Our study highlights the large effect that experiences of sexual violence in adolescence can have on mental health, especially for girls, and suggests that eliminating experiences of sexual violence at this crucial developmental stage would result in substantial reductions in mental health problems and narrow the observed gender gap in mental ill health at this age. The findings of this study, alongside previous evidence, highlight the need to consider this common and important, yet often understudied, gendered risk factor more seriously and urgently in adolescent mental health research and policy making. The widespread nature of these experiences emphasises the need to acknowledge them in clinical practice with this age group and ensure appropriate support is provided to affected individuals.

We aimed to estimate the effect of sexual violence experienced in mid-adolescence on mental health outcomes—depressive symptoms, self-harm, and attempted suicide—in a longitudinal cohort study. Although in many health contexts, randomised controlled trials are the gold standard for establishing causal effects, such studies are ethically impermissible for sexual violence experiences. We used propensity score matching (PSM),^21^ in which each individual from the treatment grou§p is matched to an individual in the control group on the basis of similarity of prespecified criteria. Due to the gendered nature of sexual violence experiences and the different salience of these experiences in girls and boys,^18^ we also aimed to stratify the analyses by gender. We hypothesised that adolescents who experienced sexual violence would have worse subsequent mental health, and that these effects would be robust to confounder adjustment. Furthermore, we aimed to predict the reduction in adverse mental health outcomes if sexual violence during mid-adolescence were to be successfully eliminated via necessary changes in policy and society, by estimating the population-attributable fraction (PAF).

## Methods

### Study design and participants

In this longitudinal study, we used data from a longitudinal cohort, including early life factors and other possible confounders, to estimate the effects of sexual violence on the mental health of mid-adolescents using two approaches. The first approach involved multivariable regression with adjustment for a wide range of relevant confounders. The second approach involved a pseudo-experimental design with a group who experienced sexual violence and a matched control group who had not (appendix p 2).

**Figure 1:**
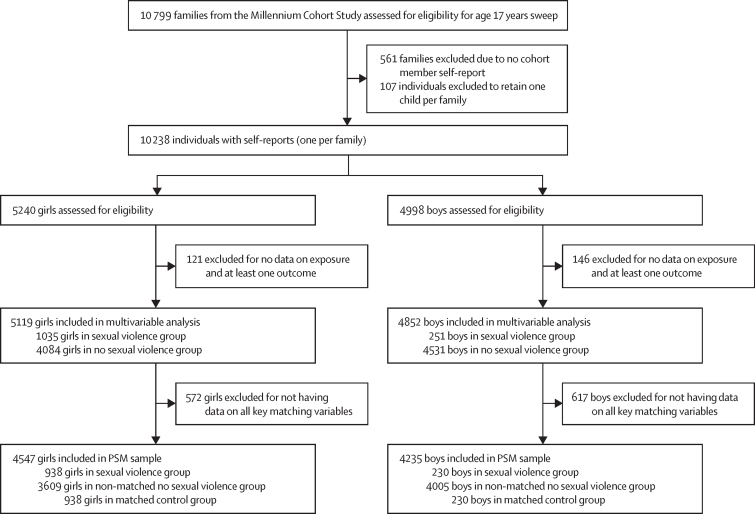
Flow diagram of the participants included in the multivariable and PSM analyses Girls were born female or identified as female. Boys were born male and did not identify as female. Sexual violence was any sexual assault or unwelcomed sexual approach in the 12 months before age 17 years. PSM=propensity score matching

Data from the Millennium Cohort Study (MCS), a national birth cohort study of 19 243 families in the UK who had a child born between 2000 and 2002, were used.^22^ Our main analysis focused on data from the sweep at age 17 years (2018–19; one child per family selected at random from the approximately 100 families with more than one cohort member), with most confounding and matching data from the previous sweep at age 14 years (2015–16).^22^ Data on other confounders were from earlier sweeps at ages 3, 5, 7, and 11 years, as relevant. For the multivariable analyses, participants had to have information available on whether they had experienced at least one type of sexual violence and on one mental health outcome at age 17 years. For the pseudo-experimental design with the matched control group, the included participants were also required to have data on all the key matching variables (figure 1). Ethical approvals for each sweep of the MCS were received from relevant national research ethics committees (appendix p 5). The MCS specifies that young people gave verbal consent.

The MCS study protocol is available online

### Procedures

Sexual violence was assessed using two questions from the self-reported questionnaire that participants completed at age 17 years. Participants were asked about their experiences of sexual assault and unwelcomed sexual approach in the previous 12 months with dichotomous (ie, yes or no) responses. We created a binary sexual violence variable by combining information from these items, but we also analysed these two variables separately.

Psychological distress at age 17 years was assessed using the Kessler Psychological Distress Scale (K6), a six-item, self-administered questionnaire that measures distress over the previous 30 days. Items refer to both depressive and anxiety symptoms and are rated on a 5-point Likert scale (with 0 being none of the time and 4 being all of the time). A total score was obtained by summing the items, with scores of 13 or above indicating high distress.^23^ The K6 measure was selected because it is the main measure capturing symptoms of common mental health conditions in the MCS cohort. The K6 measure was included in the cohort because it is a well validated, short measure of population psychological distress with established clinical thresholds that is used in many countries. Further details of the measure and its use are in the appendix (p 3).

Self-harm was assessed at age 17 years by asking participants whether they hurt themselves on purpose in the previous 12 months by cutting, burning, or bruising, or by taking an overdose, pulling out their hair, or hurting themselves in other ways. Responses to these questions were dichotomous (ie, yes or no). A binary variable was created indicating self-harm in the previous 12 months if participants responded affirmatively to any of these questions.

Attempted suicide was assessed at age 17 years via dichotomous responses (ie, yes or no) to the question: “have you ever hurt yourself on purpose in an attempt to end your life?”.

We chose matching and confounding factors in line with previous literature that indicated they were probably relevant confounders of sexual violence and mental health. These matching and confounding factors included previous mental ill health (depressive symptoms and self-harm at age 14 years), family and sociodemographic factors (ethnicity, sexuality, parent education, family income, number of siblings, and number of parents or carers), puberty-related variables (age of menarche for girls, pubertal status, early sexual activity, or previous sexual violence reported at age 14 years), interpersonal relationships (relationship status, peer relationships, and bullying); and health-related variables (BMI, risky behaviours [smoking, alcohol use, and drug use], missing school without parents’ permission, disability, and life satisfaction). Detailed information regarding the justification and measurement of these variables is in the appendix (pp 3–4).

### Statistical analyses

For missing information within the analytical sample, multiple imputation with chained equations was used, with ten imputations for exposure, outcomes, and confounders, using the mi impute chained command. We used a combined sampling and non-response weight provided with the MCS data at age 17 years to account for sampling design and attrition from previous sweeps.

We conducted linear regressions for the continuous outcome (psychological distress) and Poisson regressions for the binary outcomes (high psychological distress, self-harm, and attempted suicide), stratified by gender.

For each outcome we began with an unadjusted model, which was followed by models with increasing amounts of adjustment in the following order: previous mental ill health (depressive symptoms and self-harm at age 14 years); sociodemographic characteristics (ie, ethnicity, sexuality, parent education, family income, number of siblings, and number of parents or carers); puberty-related variables (age of menarche for girls, pubertal status, early sexual activity, and sexual violence reported at age 14 years); interpersonal relationships (ie, relationship status, peer relationships, and bullying); and lastly, in the fully adjusted model, we added health-related variables (BMI, risky behaviours, missing school without parents’ permission, disability, and life satisfaction; appendix pp 3–4). The complex survey design of the MCS was accounted for, and sample and attrition weights were used.

We used PSM^21^ to generate a control group that was matched to the group who had experienced sexual violence on key variables (appendix p 5) using psmatch2^24^ and a randomly ordered sample. We used optimal full matching to identify the closest match in the control group for each participant in the sexual violence group (1:1 ratio) on the basis of the matching variables. The quality of the matching was assessed using covariate imbalance testing and presented using graphs. Once matched, simple unweighted linear and Poisson regressions were estimated to investigate the effect of group membership (ie, the presence or absence of sexual violence) on mental health outcomes. As a sensitivity analysis, matched control groups selected by different matching variables were created to examine whether findings varied with differences in the matching variables used. We also conducted a sensitivity analysis with the PSM sample in which we excluded participants who reported sexual violence before age 14 years, to examine whether the estimates varied when focusing on the group who did not report previous sexual violence before age 14 years.

As a final robustness check, we combined both approaches and merged the matched PSM groups with the full imputed sample to adjust the analysis in the matched sample for all the additional confounders that were not accounted for by matching.

As an exploratory analysis, we conducted multivariate analyses separately for unwelcome sexual approach and sexual assault to evaluate whether the observed effect sizes were similar for these exposures when considered separately.

Furthermore, we estimated PAFs to provide an interpretable estimate of effect sizes^25^ using punaf^26^ for the overall sexual violence exposure, and for unwelcome sexual approach and sexual assault, separately. The PAF defines the fraction of all cases of a particular disease or adverse condition (eg, mental ill health) that is attributable to a specific exposure (eg, sexual violence), and can be used to estimate the proportion of the population who would have a specific outcome in a hypothetical scenario in which the exposure is null.

All analyses were conducted using Stata, version 17.

### Role of the funding source

The funder of the study had no role in study design, data collection, data analysis, data interpretation, or writing of the report.

## Results

Of 10 238 participants with self report in the MCS sweep at age 17 years, we included 9971 individuals in the full analysis sample (figure 1). Of 5119 girls and 4852 boys included in the multivariable analysis, 4138 (80·8%) girls and 3925 (80·9%) boys were White, 561 (11·0%) girls and 528 (10·9%) boys were Asian, 181 (3·5%) girls and 178 (3·7%) boys were Black, 159 (3·1%) girls and 136 (2·8%) boys were of mixed race or ethnicity, and 80 (1·6%) girls and 84 (1·7%) boys were of another race or ethnicity. One person in this cohort had missing ethnicity information.

**Figure 2:**
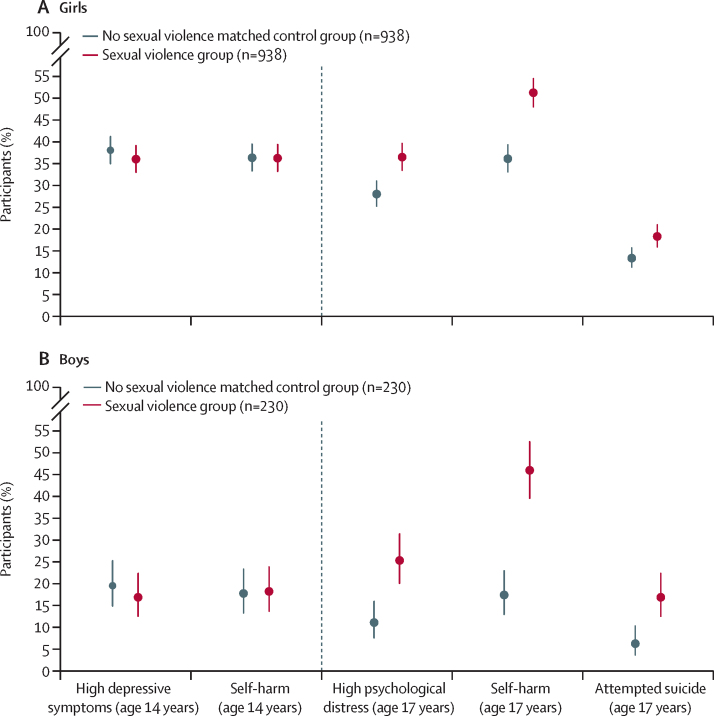
Prevalence of high depressive symptoms, self-harm, and attempted suicide at age 14 years and 17 years in adolescents who experienced sexual violence compared with those who did not Prevalence data from propensity score matched groups. The group who reported experiences of sexual violence at age 17 years were 1:1 matched to identify a control group who did not report such experiences but were similar on a range of other characteristics; error bars show 95% CI. Sexual violence was any sexual assault or unwelcomed sexual approach in the 12 months before age 17 years. Depressive symptoms at age 14 years were assessed using the Short Mood and Feelings Questionnaire and psychological distress at age 17 years was assessed using Kessler Psychological Distress K6 scale. At age 14 years, high depressive symptoms were assessed for prevalence in the past 2 weeks and self-harm was assessed for prevalence in the past 12 months. At age 17 years, high psychological distress was assessed for prevalence in the past 30 days, self-harm was assessed for prevalence in the past 12 months, and attempted suicide was assessed for lifetime prevalence.

At age 17 years, 1035 (20·2%) of 5119 girls in the full analysis sample had reported experiencing any sexual violence in the previous 12 months (269 [5·3%] experienced assault and 991 [19·4%] experienced unwelcome sexual approach; table 1). 263 (5·4%) of 4852 boys had experienced any sexual violence (50 [1·0%] experienced sexual assault and 251 [5·2%] experienced unwelcome sexual approach). The corresponding data for the PSM sample are shown in the appendix (p 8). Venn diagrams (appendix p 7) show overlap between the two types of sexual violence and highlight that most participants who reported assault also reported unwelcome sexual approaches. Figure 2 shows the similar rates of mental health outcomes at age 14 years for the matched groups, followed by the rates at age 17 years, which were higher for girls and boys who experienced sexual violence compared with the matched control group.

**Table 1:** Descriptive analysis of mental health measures at age 17 and age 14 years for girls and boys in the full analysis sample

		**Girls**			**Boys**		
		Total (n=5119)	Sexual violence group (n=1035)	No sexual violence group (n=4084)	Total (n=4852)	Sexual violence group (n=263)	No sexual violence group (n=4589)
**Mental health at age 17 years**							
Psychological distress in the past 30 days, using K6		8·41 (0·18; 8·06–8·75)	10·92 (0·25; 10·43–11·42)	7·75 (0·21; 7·33–8·17)	6·17 (0·12; 5·95–6·40)	9·72 (0·34; 9·04–10·39)	5·97 (0·12; 5·73–6·20)
High psychological distress (K6 score ≥13) in the past 30 days							
	No	3974 (77·4%; 74·9–79·7)	644 (60·0%; 55·0–65·3)	3330 (81·8%; 79·0–84·3)	4376 (89·8%; 88·0–91·4)	192 (77·2%; 67·5–84·6)	4184 (90·5%; 88·7–92·1)
	Yes	1141 (22·6%; 20·3–25·1)	390 (40·0%; 34·7–45·0)	751 (18·2%; 15·7–21·0)	474 (10·2%; 8·6–12·0)	71 (22·8%; 15·4–32·5)	403 (9·5%; 7·9–11·3)
Self-harm in the past 12 months							
	No	3520 (71·7%; 69·3–73·9)	475 (47·3%; 42·8–51·9)	3045 (77·6%; 75·1–80·0)	3938 (79·9%; 77·3–82·3)	134 (44·3%; 31·4–58·1)	3798 (81·9%; 79·5–84·1)
	Yes	1439 (28·3%; 26·1–30·7)	506 (52·7%; 48·1–57·2)	930 (22·4%; 20·0–24·9)	826 (20·1%; 17·7–22·7)	116 (55·7%; 41·9–68·6)	710 (18·1%; 15·9–20·5)
Attempted suicide in lifetime							
	No	4437 (89·4%; 87·8–90·7)	791 (78·2%; 73·0–82·5)	3646 (92·1%; 90·7–93·3)	4551 (95·8%; 94·8–96·6)	207 (85·1%; 77·0–90·7)	4344 (96·4%; 95·4–97·1)
	Yes	509 (10·6%; 9·3–12·2)	188 (21·8%; 17·5–27·0)	321 (7·9%; 6·7–9·3)	209 (4·2%; 3·4–5·2)	44 (14·9%; 9·3–23·0)	165 (3·6%; 2·9–4·6)
**Mental health at age 14 years**							
Depressive symptoms in the past 2 weeks, using SMFQ		7·10 (0·14; 6·82–7·38)	9·60 (0·36; 8·88–10·31)	6·44 (0·14; 6·16–6·72)	4·05 (0·11; 3·84–4·26)	5·55 (0·47; 4·62–6·47)	3·97 (0·11; 3·75–4·19)
High depressive symptoms (SMFQ score ≥12) in the past 2 weeks							
	No	3559 (77·5%; 75·9–79·1)	609 (64·1%; 59·9–68·1)	2990 (81·1%; 79·4–82·7)	3972 (91·2%; 89·8–92·4)	196 (86·0%; 80·3–90·2)	3776 (91·5%; 90·0–92·8)
	Yes	1038 (22·5%; 20·9–24·1)	344 (35·9%; 31·8–40·1)	694 (18·9%; 17·3–20·6)	352 (8·8%; 7·6–10·2)	41 (14·0%; 9·8–19·7)	311 (8·5%; 7·2–10·0)
Self-harm in the past 12 months							
	No	3648 (77·9%; 76·1–79·6)	605 (63·8%; 59·2–68·1)	3043 (81·6%; 79·8–83·3)	3991 (91·0%; 89·2–92·4)	190 (82·6%; 76·4–87·2)	3801 (91·4%; 89·6–92·9)
	Yes	993 (22·1%; 20·4–23·9)	344 (36·2%; 31·9–40·8)	649 (18·4%; 16·7–20·2)	356 (9·0%; 7·6–10·8)	49 (17·4%; 12·8–23·2)	307 (8·6%; 7·1–10·4)

Data are mean (SE; 95% CI) for continuous variables or n (%; 95% CI) for dichotomous variables. These data are weighted using a combined sample and non-response weight for the analytical sample. K6= Kessler Psychological Distress Scale. SMFQ=Short Mood and Feelings Questionnaire.

Multivariable regressions in the full analysis sample (ie, multiple imputation) with increasing amounts of adjustment indicate that individuals who reported experiencing sexual violence in the 12 months before age 17 years had worse mental health outcomes at age 17 years than individuals who did not (table 2). Some confounder adjustments had a substantial effect on the estimates, whereas additional adjustments affected the estimates less in comparison (table 2). In the final adjusted model, individuals who experienced sexual violence had higher mean psychological distress (mean difference 2·09 [95% CI 1·51–2·68] in girls and 2·56 [1·59–3·53] in boys), a higher risk of having high psychological distress girls (risk ratio [RR] 1·65 [95% CI 1·37–2·00] in girls and 1·55 [1·00–2·40] in boys), a higher risk of self-harm (RR 1·79 [1·52–2·10] in girls and 2·16 [1·63–2·84] in boys), and a higher risk of attempting suicide (RR 1·75 [1·26–2·41] in girls and 2·73 [1·59–4·67] in boys) than individuals who did not experience sexual violence (table 2).

**Table 2:** Mean differences and RRs for sexual violence predicting mental health outcomes from a range of modelling approaches

	Psychological distress in the past 30 days*, mean difference (95% CI)		**High psychological distress in the past 30 days, RR (95% CI)**		**Self-harm in the past 12 months, RR (95% CI)**		**Attempted suicide ever, RR (95% CI)**	
	Girls	Boys	Girls	Boys	Girls	Boys	Girls	Boys
**Multivariable regressions**								
Unadjusted	3·16 (2·52–3·81)	3·75 (3·04–4·46)	2·18 (1·79–2·66)	2·41 (1·62–3·59)	2·32 (1·99–2·71)	3·09 (2·38–4·03)	2·67 (2·01–3·54)	4·17 (2·54–6·84)
Adjustment 1	2·14 (1·47–2·81)	2·11 (2·18–4·05)	1·67 (1·36–2·05)	1·87 (1·23–2·84)	1·90 (1·60–2·25)	2·62 (1·90–3·62)	1·84 (1·32–2·56)	3·00 (1·84–4·88)
Adjustment 2	2·02 (1·37–2·67)	2·59 (1·61–3·56)	1·62 (1·32–1·98)	1·58 (1·02–2·45)	1·79 (1·52–2·09)	2·23 (1·63–2·74)	1·72 (1·22–2·41)	2·80 (1·66–4·72)
Adjustment 3	2·10 (1·45–2·75)	2·57 (1·60–3·54)	1·65 (1·35–2·02)	1·57 (1·01–2·44)	1·81 (1·54–2·12)	2·22 (1·72–2·88)	1·74 (1·24–2·43)	2·60 (1·53–4·41)
Adjustment 4	2·07 (1·44–2·71)	2·64 (1·69–3·59)	1·64 (1·35–1·99)	1·64 (1·08–2·50)	1·80 (1·54–2·11)	2·24 (1·73–2·88)	1·75 (1·26–2·44)	2·60 (1·55–4·36)
Fully adjusted model	2·09 (1·51–2·68)	2·56 (1·59–3·53)	1·65 (1·37–2·00)	1·55 (1·00–2·40)	1·79 (1·52–2·10)	2·16 (1·63–2·84)	1·75 (1·26–2·41)	2·73 (1·59–4·67)
**Matched samples**								
PSM (all matching variables)	1·35 (0·88–1·81)	2·73 (1·84–3·63)	1·30 (1·14–1·49)	1·97 (1·31–2·93)	1·42 (1·28–1·58)	1·92 (1·47–2·52)	1·37 (1·11–1·70)	2·28 (1·33–3·93)
PSM (all variables minus sexual identity, pubertal status, parental education, and sexual violence before age 14 years)	1·66 (1·19–2·14)	3·29 (2·41–4·16)	1·35 (1·18–1·54)	2·68 (1·70–4·23)	1·58 (1·41–1·77)	2·18 (1·64–2·92)	1·44 (1·16–1·79)	4·28 (2·12–8·65)
PSM (all variables minus high psychological distress, self-harm at age 14 years, and sexual violence before age 14 years)	2·37 (1·92–2·83)	3·53 (2·67–4·38)	1·72 (1·48–2·00)	3·11 (1·91–5·04)	1·82 (1·62–2·06)	2·24 (1·67–3·00)	2·17 (1·69–2·80)	4·83 (3·04–18·97)
**Matched sample with multivariable confounder adjustment**								
Adjusted regression analysis	1·35 (0·92–1·79)	2·54 (1·68–3·40)	1·31 (1·16–1·49)	1·87 (1·26–2·80)	1·41 (1·27–1·57)	1·83 (1·40–2·40)	1·37 (1·11–1·68)	2·36 (1·37–4·08)
**Multivariable adjusted regression by sexual violence type**								
Unwelcome sexual approach	2·03 (1·44–2·63)	2·61 (1·61–3·60)	1·49 (1·34–1·65)	1·88 (1·44–2·45)	1·56 (1·44–1·70)	1·99 (1·67–2·37)	1·44 (1·21–1·71)	2·54 (1·72–3·76)
Sexual assault	2·32 (1·49–3·15)	3·82 (1·82–5·81)	1·46 (1·27–1·68)	1·87 (1·11–3·13)	1·57 (1·41–1·75)	2·09 (1·56–2·80)	1·72 (1·40–2·11)	2·63 (1·38–5·01)
Unwelcome sexual approach only†	1·51 (1·16–1·86)	2·05 (1·49–2·60)	1·45 (1·29–1·64)	1·80 (1·36–2·38)	1·49 (1·35–1·65)	1·87 (1·54–2·26)	1·27 (1·03–1·57)	2·33 (1·55–3·53)

The models had sequentially increasing amounts of adjustment. Adjustment 1 was for previous mental ill health (depressive symptoms and self-harm up to 14 years of age). Adjustment 2 added sociodemographic or economic characteristics (ethnicity, parental education, family income, sexual identity, single parent household, and number of siblings). Adjustment 3 added puberty-related characteristics (pubertal status, age of menarche [only girls], early sexual activity, and experience of sexual violence up to 14 years of age). Adjustment 4 added interpersonal characteristics (relationship status, peer relationships, and bullying). The fully adjusted model added health-related characteristics (BMI, risky behaviours, missing school without parents’ permission, disability, and life satisfaction). PSM=propensity score matching. RR=risk ratio.

* This is a continuous measure.

† Individuals who reported unwelcome sexual approach and no sexual assault.

Complete case analyses are in the appendix (p 9) and show broadly similar results with bias in the expected direction (ie, underestimation) due to attrition being predicted by socioeconomic disadvantages, poorer health, and other disadvantaging factors.

The percentages and means for exposure, outcomes, and each of the key matching variables were similar between the full sample and the PSM sample (appendix pp 11–15). The quality of the matching was assessed through evaluation of the percentage of bias for the matching variables (appendix pp 19–20), which was found to be low (<10%) for all variables except previous sexual violence before 14 years of age in girls, for which bias was 10·3%.

**Figure 3:**
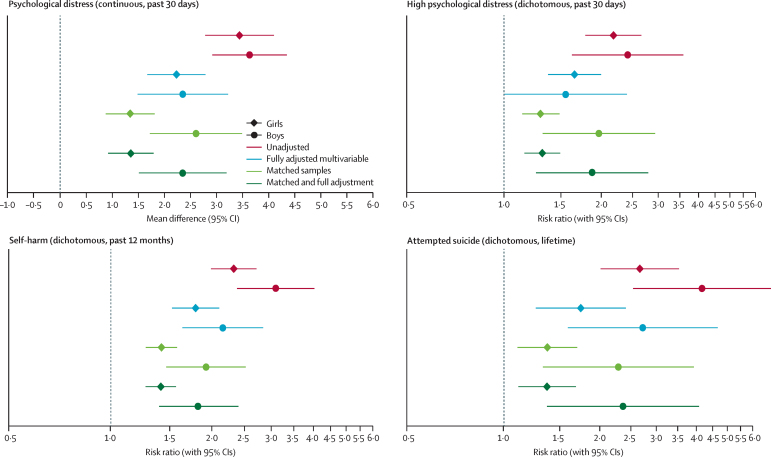
Multivariable regression analyses and PSM analyses estimating the effect of sexual violence on psychological distress, self-harm, and attempted suicide at age 17 years, according to extent of adjustment and matching Error bars show 95% CI. Sexual violence was any sexual assault or unwelcomed sexual approach in the 12 months before age 17 years. Psychological distress was analysed as both a continuous and dichotomous variable; self-harm and attempted suicide were analysed as dichotomous variables. The dotted line is a reference line for the interpretation of the coefficients. A value on the right side of the line indicates worse outcomes for those who experience sexual violence and an estimate on the left of the line would indicate a better outcome. PSM=propensity score matching. RR=risk ratio.

In the PSM-matched samples, the group who experienced sexual violence had a higher mean score of psychological distress than the matched control group (mean difference 1·35 [95% CI 0·88–1·81] in girls and 2·73 [1·84–3·63] in boys; table 2). Furthermore, the group who experienced sexual violence had a greater risk of high distress (1·30 [1·14–1·49] in girls and 1·97 [1·31–2·93] in boys), self-harm (1·42 [1·28–1·58] in girls and 1·92 [1·47–2·52] in boys), and attempted suicide (1·37 [1·11–1·70] in girls and 2·28 [1·33–3·93] in boys) than the matched control group (table 2; figure 3).

In our sensitivity analyses for the PSM sample using different matching variables to evaluate the best match and the difference in their estimates, we found that different effect sizes were estimated when different matching variables were used; estimates were often higher when the matching excluded a few variables than when the matching was conducted with all variables (table 2). Sensitivity analyses matching a sample excluding participants who had reported experiences of sexual violence before they were aged 14 years show similar effect sizes to the analyses in the full sample (appendix p 19).

Analyses combining the matching and the full confounder adjustment resulted in similar effect sizes (table 2), suggesting that the estimates are likely to be robust and any unmeasured confounder would have to have a fairly strong influence to reduce the observed effects.

Additionally, in multivariate analyses for unwelcomed sexual approach, sexual assault, and unwelcome sexual approach only (excluding the subset who also experienced sexual assault), we found larger effects of reporting sexual assaults than only reporting unwelcome sexual approach for psychological distress and self-harm in boys and psychological distress and attempted suicide in girls (table 2).

**Figure 4:**
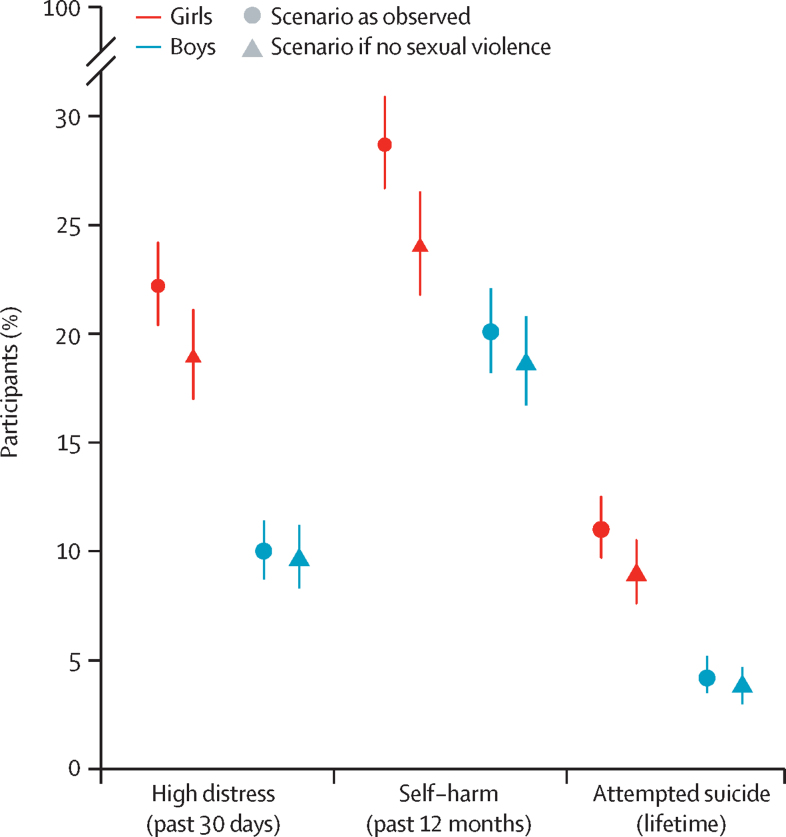
Observed prevalence of mental health outcomes at age 17 years versus predicted prevalence in a hypothetical counterfactual scenario in which no sexual violence occurred Error bars show 95% CI. Prevalence in the hypothetical scenario of no sexual violence was estimated using the population attributable fraction. Sexual violence was any sexual assault or unwelcomed sexual approach in the 12 months before age 17 years.

Our PAF estimates showed that in a hypothetical scenario in which girls did not experience any sexual violence, the prevalence of serious mental health problems at age 17 years would be 14·0–18·7% lower than in the observed scenario; the prevalence of attempted suicide in girls would be 9·1% (95% CI 7·8–10·8) in a scenario without sexual violence compared with the observed prevalence of 11·0% (10·0–12·7), and that of high psychological distress would be 19·5% (17·7–21·5) compared with the observed 22·6% (21·0–24·4; figure 4). For boys, the prevalence of serious mental health problems would be 3·7–10·5% lower in a scenario in which they did not experience any sexual violence; the prevalence of attempted suicide would be 3·9% (3·2–4·7) in a scenario without sexual violence compared with the observed prevalence of 4·3% (3·6–5·2), and that of high psychological distress would be 9·8% (8·5–11·4) compared with the observed 10·2% (8·9–11·7; figure 4)**.**

## Discussion

In our sample of individuals from the UK Millenium Cohort Study who reported experiencing sexual violence in the 12 months before age 17 years reported worse mental health outcomes at age 17 years than adolescents who did not. These effects persisted even after accounting for previous depressive symptoms and self-harm, and a wide range of relevant confounders, and were robust to multiple methodological approaches and sensitivity checks.

Because this study used data from a national cohort study, the reported effect sizes can be considered in a public health context. Due to the large effect sizes for all the analysed mental health outcomes (RR >1·30), these findings could have important real-world implications. Our PAF findings, assuming this effect is causal and robust to further potential confounding (appendix p 20), suggest that the absence of experiences of sexual violence during mid-adolescence would result in a 14·0–18·7% relative reduction in the prevalence of adverse mental health outcomes in girls and a 3·7–10·5% relative reduction in boys, thus also substantially narrowing the gender gap in mental health disorders reported at this age.^2^

Sexual violence during mid-adolescence contributes markedly to the high prevalence of mental ill health observed in adolescents, especially in girls, and actively tackling sexual violence could lead to substantial improvements in adolescent mental health. In a UK report published in 2021, estimates from school populations showed high levels of sexual violence, with 79% of the surveyed girls reporting sexual assault of any kind.^27^ The same report highlighted the perceived inadequacy of school-based sex education. However, peer-on-peer sexual violence in schools could be tackled with appropriate school-based intervention and education.^28^ Effective intervention and education is even more relevant given the fact that many adolescents do not report such experiences due to the stigma around it.^29^ Other avenues for intervention include within families and interpersonal relationships via social services, primary care, and other community-based services.^30^

Biological factors, social factors, familial factors, and ascertainment biases have been attributed as causes for the emergent gender gap in depression and other mental health difficulties in adolescence.^1^, ^3^ For example, pressures around body image and academic achievements are often cited as important gendered risk factors. Our findings clearly highlight the role of sexual violence, which is disproportionally experienced by girls, as an important and preventable driver of the gender gap in adolescent mental health.

There were some limitations to our study. First, information on mental health and past experiences of sexual violence were collected at the same time in the assessment at age 17 years, and there is some evidence that mental ill health affect the recall and reporting of these adverse experiences.^31^ The question on self-harm with suicidal intention at age 17 years asked the individual about their lifetime prevalence of self-harm and suicidal intention, and although we controlled for self-harming before 14 years of age, there are issues around timing and temporal precedence of exposure and outcome that we cannot resolve. In addition, reported prevalence of sexual violence, albeit high, was lower than the prevalence suggested by some other data sources.^5^, ^27^ Hence, it is possible that the association we observed was overestimated because adolescents with fewer mental health difficulties might not have reported their sexual violence experiences. However, it is also possible that under-reporting might have resulted in a downward bias and an underestimation of effects. Future studies with more detailed and better quality longitudinal follow-up data will allow better understanding of these findings. However, the under-reporting of incidences of sexual violence will probably remain an issue. Second, there are limitations of the questions asked. For example, participants were asked about sexual violence experiences in the previous 12 months, meaning that incidences before this period would not have been reported. The questions about sexual violence were dichotomous, which meant it was not possible to investigate important aspects such as severity, frequency, and perpetrator. Less severe experiences, such as harassment, that are experienced constantly could have a substantial effect. The sexual violence measures used also did not include the full range of these potential experiences. For example, harassment via online platforms or over text was not asked about and young people might not have reported these experiences in the unwelcome sexual approach question. More specific questions are needed to fully understand the effects of sexual violence at this age. Third, potential unmeasured confounders (eg, parental neglect or violence between parents) not captured in this study might attenuate the observed effects. Despite PSM being a useful technique in observational studies when RCTs are not possible, and although matching can improve efficiency and precision, it is limited in reducing confounding by unobserved variables. Future research should aim to triangulate these findings using alternative approaches to assess the effect of sexual violence on mental health.

Most research and policy focussed on sexual violence is directed to either child sexual abuse or interpersonal violence within relationships in older adolescents and adults. However, a third category of violence—ie, violence experienced in community settings on the streets, by peers, in schools, and online—is also relevant and especially salient as children transition into adolescence and adulthood. The scarcity of population-based datasets that include detailed measures on the perpetrators, frequency, severity, types, and settings of sexual violence severely restricts the capacity of research to estimate the real consequences of these different experiences, which would help to target appropriate policy and support. The inadequacy of the data highlights the importance of formulating more comprehensive research questions about sexual violence experiences and the need to gather more data on such experiences in adolescence. Importantly, sexual violence is known to be widespread in adulthood as well,^32^ thus suggesting a continuation and potential worsening of mental health sequelae of these experiences. Because sexual violence is a global phenomenon, it is imperative to increase efforts to understand its role in the mental health of adolescent girls while also promoting the development of effective strategies both to prevent sexual violence and to mitigate its subsequent effects.

The implications of our findings are clear and multifold. A reduction in sexual violence would probably lead to a decrease in mental health problems, which could, in turn, reduce the societal costs not only for the individuals affected, but also for their families and wider society.^33^ Starting early, interventions aimed at educating young people in schools with regard to sexual violence are one actionable avenue.^34^ Moreover, more attention should be paid at the societal scale by policy makers to reduce the general societal tolerance and permissiveness currently shown for sexual violence.^35^ The low conviction rates for perpetrators have severe effects on the mental health of individuals who experience sexual violence,^36^ and erodes trust in the law enforcement systems that are meant to protect individuals who experience sexual violence. Changes to legal systems and law enforcement might reduce risks—eg, by tackling the existing policies regarding prosecutions and creating more sexual violence deterrents. Experiences of sexual violence are traumatic in nature, and this must be acknowledged in the responses and support offered.^37^ Furthermore, reducing victim blaming, which is prevalent in law enforcement and legal systems, is also important.^38^ The effect sizes observed also highlight the urgent need for better tailored and targeted support for victims, to try to mitigate the long-term mental health effects of sexual violence.

This study estimated the effect of sexual violence experienced in mid-adolescence on mental health outcomes using a robust methodology in a longitudinal, population-based cohort study. The strengths of our study include that it used a population-based cohort, had longitudinal data, and had adjustment for a rich set of important factors that might confound the association between sexual violence and adverse mental health outcomes. These strengths, combined with methods that aim to improve causal inference, help to provide robust estimates of the effects of sexual violence in adolescence on mental health. Our findings highlight the large burden of sexual violence experienced in mid-adolescence and stress the importance of reducing these experiences, especially for girls. Addressing this risk factor would contribute substantially to closing the gender gap in mental health outcomes that emerges in adolescence. The findings of this study emphasise the need to consider this common and important, yet often gendered and understudied, risk factor more seriously and urgently in adolescent mental health research, clinical practice, and policy making.

## Data sharing

The Millennium Cohort Study data are available to use by researchers via the UK Data Service (https://ukdataservice.ac.uk/).

For the **study protocol** see https://cls.ucl.ac.uk/cls-studies/millennium-cohort-study/mcs-age-17-sweep/]

## Declaration of interests

We declare no competing interests.
